# Everolimus and Malignancy after Solid Organ Transplantation: A Clinical Update

**DOI:** 10.1155/2016/4369574

**Published:** 2016-10-11

**Authors:** Hallvard Holdaas, Paolo De Simone, Andreas Zuckermann

**Affiliations:** ^1^Section of Nephrology, Department of Transplant Medicine, Oslo University Hospital, Rikshospitalet, Postboks 4950 Nydalen, 0424 Oslo, Norway; ^2^Hepatobiliary Surgery & Liver Transplantation, Azienda Ospedaliero-Universitaria Pisana, 5412 Pisa, Italy; ^3^Department of Cardiac Surgery, Medical University of Vienna, Währinger Gürtel 18-20, 1090 Vienna, Austria

## Abstract

Malignancy after solid organ transplantation remains a major cause of posttransplant mortality. The mammalian target of rapamycin (mTOR) inhibitor class of immunosuppressants exerts various antioncogenic effects, and the mTOR inhibitor everolimus is licensed for the treatment of several solid cancers. In kidney transplantation, evidence from registry studies indicates a lower rate of* de novo* malignancy under mTOR inhibition, with some potentially supportive data from randomized trials of everolimus. Case reports and small single-center series have suggested that switch to everolimus may be beneficial following diagnosis of posttransplant malignancy, particularly for Kaposi's sarcoma and nonmelanoma skin cancer, but prospective studies are lacking. A systematic review has shown mTOR inhibition to be associated with a significantly lower rate of hepatocellular carcinoma (HCC) recurrence versus standard calcineurin inhibitor therapy. One meta-analysis has concluded that patients with nontransplant HCC experience a low but significant survival benefit under everolimus monotherapy, so far unconfirmed in a transplant population. Data are limited in heart transplantation, although observational data and case reports have indicated that introduction of everolimus is helpful in reducing the recurrence of skin cancers. Overall, it can be concluded that, in certain settings, everolimus appears a promising option to lessen the toll of posttransplant malignancy.

## 1. Introduction

Malignancy after solid organ transplantation is substantially more frequent than in the general population [1–3] and remains a major cause of posttransplant mortality [4, 5]. One large analysis recently reported a twofold increase in risk after transplantation [4], and for some virus-related cancers, such as Kaposi's sarcoma and non-Hodgkin lymphoma, the increase in risk is far higher [2, 3, 6]. Oncogenesis is promoted in transplant patients receiving maintenance immunosuppressive therapy due to impaired immune surveillance and a more permissive environment for viral replication. It is becoming clear, however, that class-specific effects are important as well as the overall intensity of immunosuppression [7].

One of the best-documented associations between immunosuppression and risk of malignancy is for the calcineurin inhibitor (CNI) class of drugs. CNI therapy has been shown to increase the risk of malignancy after kidney [8–10], liver [11], and heart [12–14] transplantation in a dose-dependent manner. It is unclear how much of this effect is due to high intensity of immunosuppression under CNI therapy or to specific CNI-related effects which promote oncogenesis, such as stimulation of transforming growth factor beta (TGF-*β*) [8] and increased production of proangiogenic vascular endothelial growth factor (VEGF) [15]. In contrast, the mammalian target of rapamycin (mTOR) inhibitor class exerts various antioncogenic effects. Regulatory genes for the mTOR pathway are lost or mutated in many cancers, leading to enhanced activation of mTOR and increased cell resistance to apoptosis [16, 17]. Disruption of mTOR activation interrupts this antiapoptotic effect and mTOR-dependent angiogenesis, both of which are essential for the development and propagation of malignant cells. mTOR inhibitors also suppress translation of mRNAs that promote carcinogenesis, such as VEGF and cyclin C1 (required for efficient cell cycles) [17]. Preclinical studies have shown mTOR inhibitors to have a potent inhibitory effect in various cancers including B-cell lymphocyte growth [18], prostate tumors [19], and renal carcinomas [20] and that they exert antimyeloma activity in multiple myeloma [21]. Furthermore, transplant recipients given an mTOR inhibitor require lower CNI doses or may be maintained on a CNI-free regimen, so antioncogenic effects of the mTOR inhibitor may be enhanced by reduced long-term CNI exposure.

The mTOR inhibitor everolimus is licensed for the prophylaxis of allograft rejection in combination with reduced-exposure CNI in adult kidney transplant patients at low or moderate immunologic risk and in liver transplant recipients. Notably, however, everolimus is also licensed for the treatment of several malignancies including advanced metastatic renal cell cancer [22, 23], gastroenteropancreatic neuroendocrine tumor [24], and subependymal giant cell astrocytoma [25]. Promising results have also been published for everolimus in the treatment of relapsed or refractory multiple myeloma [26], biliary tract cancer [27], non-Hodgkin lymphoma [28], certain breast cancers [29–31], Kaposi's sarcoma [32], and other malignancies [25, 33–35]. The combination of immunosuppressive efficacy with therapeutic antioncogenic effects has raised the question of whether everolimus-based immunosuppressive regimens could help to prevent and manage posttransplant malignancies. Drawing firm conclusions is challenging, however. First, the relative rarity and long development time [2] of posttransplant malignancy mean that large patient populations must be followed up over an extended period to obtain adequate analytical power. Second, the etiology of cancer is so multifactorial that identifying the contribution of one variable is difficult.

Recent published reviews have assessed the evidence base relating to mTOR inhibitors overall [17, 36] but the two available agents, sirolimus and everolimus, are not necessarily interchangeable [37]. This article examines the available data and considers the role of everolimus in malignancy after organ transplantation from the clinician's perspective.

## 2. Methods

The PubMed database was searched with no language or time limitations. Multiple searches were performed using combinations of the following terms: transplantation, mTOR, everolimus, malignancy, neoplasm, cancer, skin cancer, Kaposi's sarcoma, hepatocellular carcinoma (HCC), and cholangiocarcinoma. The reference lists of review articles were checked manually for additional citations.

## 3. Kidney Transplantation

### 3.1. Prevention of* De Novo* Malignancies

**Figure 1:**
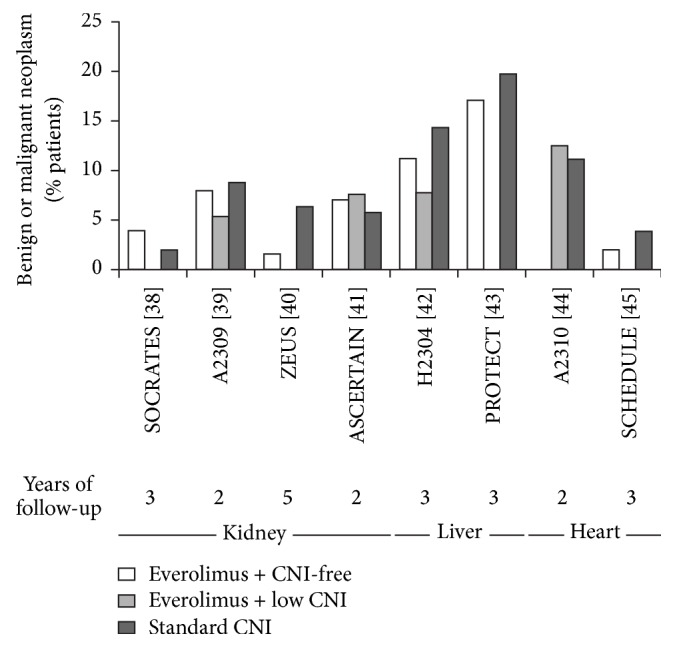
Incidence of neoplasms (benign or malignant) in randomized trials of everolimus within a CNI-free or low-CNI regimen. CNI, calcineurin inhibitor [38–45].

Prospective or retrospective analyses for risk of malignancy specifically related to everolimus after kidney transplantation are lacking. Four randomized studies of everolimus in kidney transplantation have reported rates of neoplasms after more than one year of follow-up [38–41], but it should be borne in mind that the limited duration and size of randomized trials preclude adequate power to detect a difference in rates of malignancy between immunosuppressive agents. The most valid data comes from ZEUS, the largest study to compare everolimus with CNI elimination versus a standard CNI regimen, in which patients were followed up to five years [40]. In the 232 patients who were followed up to year 5, neoplasms occurred in 1.6% of everolimus-treated patients (2/123: one benign tumor and one basal cell carcinoma) and 6.4% of the CNI group (7/109: 3 nonmalignant skin cancers and four solid tumors). Studies with shorter follow-up have not shown a difference between groups (Figure 1).

Registry analyses do not provide the analytical rigor of controlled trials but offer large numbers and longer follow-up. A relatively early analysis of 33,249 patients undergoing kidney transplantation during 1996 to 2001, censored at a maximum of 963 days' follow-up, found the relative risk of any* de novo* malignancy to be significantly lower under mTOR inhibitors versus CNI therapy [46] but dosing regimens for both classes of drug have evolved since that time and the results are not necessarily applicable to today's practice. A more recent cohort of 7,217 patients, transplanted in Italy during 1997–2009, however, also observed a significantly reduced risk (46%) for* de novo* cancer with use of mTOR inhibitor therapy compared to no mTOR inhibitors [2]. Neither study nor any other registry analysis assessed everolimus and sirolimus separately.

### 3.2. Management of Posttransplant Malignancy

Retrospective series and case reports have described outcomes following switch to everolimus-based immunosuppression following a diagnosis of malignancy. Identifying the contribution for everolimus is inevitably difficult since other interventions are also usually instituted, reflecting real-life practice. A cohort of 21 patients with malignant neoplasms who were converted to everolimus at a mean of 108 months after kidney transplantation was documented in the Argentinean Registry of Renal Transplant Recipients [47]. The malignancies included skin (7), gynecological (3), gastrointestinal (3), renal (2), prostate (1), central nervous system (1) cancers, posttransplant lymphoproliferative disease (PTLD, 2), seminoma (1), and Kaposi's sarcoma (1). All but one patient discontinued CNI therapy after starting everolimus, and in 16 cases patients underwent surgical intervention with chemotherapy or radiotherapy. No patient developed rejection or discontinued everolimus by last follow-up (mean 505 days) and no patient died from cancer during follow-up. The authors concluded that conversion to everolimus for posttransplant neoplasm is a valid therapeutic approach [47]. In another series, a single-center retrospective analysis, 25 kidney transplant patients were switched from CNI therapy to everolimus after diagnosis of malignancy: 17 had nonmelanoma skin cancer (NMSC) and the remaining eight had solid cancers [48]. In 19 of the 25 patients, low-exposure CNI therapy was continued after starting everolimus. There were no cases of rejection or increased proteinuria. Encouragingly, only two patients (8%) experienced relapse during a mean follow-up of 18 months. There was no recurrence of skin tumors and three patients with prostate cancer or Kaposi's sarcoma were in remission at last follow-up [48]. The literature also provides single case reports in which kidney transplant patients were converted to everolimus due to solid cancers including renal cell carcinoma [49] and gynecological malignancy [50] and PTLD [51], in parallel with other interventions, and achieved regression or remission.

Use of everolimus in Kaposi's sarcoma, a skin tumor of multicentric origin, is of particular interest. In some ethnic groups, it can occur in as many as 5% of kidney transplant recipients, typically developing in the first two years after transplant [52]. Expression of VEGF and other angiogenesis-related signalling proteins is upregulated in Kaposi's sarcoma lesions compared to normal skin [52]. mTOR inhibitors inhibit VEGF production and the response of endothelial cells to VEGF [15]. Reduction of immunosuppressive intensity is the first step in management, but the Kidney Disease Improving Global Outcomes (KDIGO) recommendations suggest that treatment with an mTOR inhibitor also be started [53]. This recommendation was largely based on evidence using sirolimus [54], but several case reports have described successful outcomes for Kaposi's sarcoma in kidney transplant patients following conversion to everolimus [48, 52, 55, 56] (Table 1). These cases, while limited, are consistent with the available evidence relating to management of Kaposi's sarcoma in nontransplanted individuals by everolimus alone [58–60].

NMSC is also a significant challenge in posttransplant management, estimated to affect between 6% and 7.5% of kidney transplant patients within 10 years [6, 10]. More than 90% of NMSC lesions are basal cell carcinomas (BCC) or squamous cell carcinomas (SCC) [61], with kidney transplant patients experiencing a 10-fold and 100-fold increase in risk for BCC and SCC, respectively, compared to the general population [62]. Although nonfatal, primary NMSC is generally more aggressive than in nontransplant populations and is associated with an increased risk for subsequent lesions [6] and for nonskin cancers [63]. The evidence relating to switch to sirolimus following diagnosis of NMSC is more extensive than for everolimus [64], but data is accumulating regarding intervention with everolimus. Caroti et al. have described a series of eleven kidney transplant patients who developed SCC at a median of 107 months after transplant [65]. The lesions were surgically excised and patients were switched to everolimus with low-dose cyclosporine (CsA). Steroids were continued but mycophenolate mofetil (MMF) was withdrawn or minimized. During a median follow-up of 22 months, only two cases of recurrent SCC were observed. Small case series and single case reports in the literature also point to a low rate of NMSC recurrence after introduction of everolimus (Table 2). Fernández and colleagues documented outcomes in six kidney transplant patients with recurrent skin cancer who were switched to everolimus [51]. No new skin lesions developed after everolimus replaced CNI therapy, over a minimum follow-up of six months. Low rates of recurrence were also reported over a 24-month follow-up period in five transplant recipients (including two kidney transplant patients) by Alter and colleagues after switch from CNI to everolimus [67]. Limited data from heart transplantation also points to a protective effect for everolimus in patients with skin cancers [68] (see “Heart Transplantation”). Prospective data in any organ type, however, are lacking.

## 4. Liver Transplantation

### 4.1. Prevention of* De Novo* Malignancies

Two randomized trials which included CNI-free everolimus and standard CNI treatment arms, both of which followed liver transplant patients to three years after transplant, have shown a small numerical reduction in the rate of neoplasms under everolimus versus controls [42, 43] (Figure 1), but the size of the follow-up populations (282 and 203 patients, resp.) prohibit any definite conclusions.

One large retrospective single-center analysis of liver transplants performed during 1996 to 2013 compared the incidence of new-onset posttransplant malignancies in 243 patients who were given everolimus for reasons other than malignancy versus 1,182 patients without any mTOR inhibitor treatment [69]. After a median follow-up of 1,740 days, the incidence of new-onset malignancies was 0.2% in the everolimus-treated group and 3.4% in the patients without an mTOR inhibitor. Everolimus-free immunosuppression was found to be an independent predictor for risk of malignancy. Confirmatory studies are lacking, however.

### 4.2. Management of Posttransplant Malignancy

**Figure 2:**
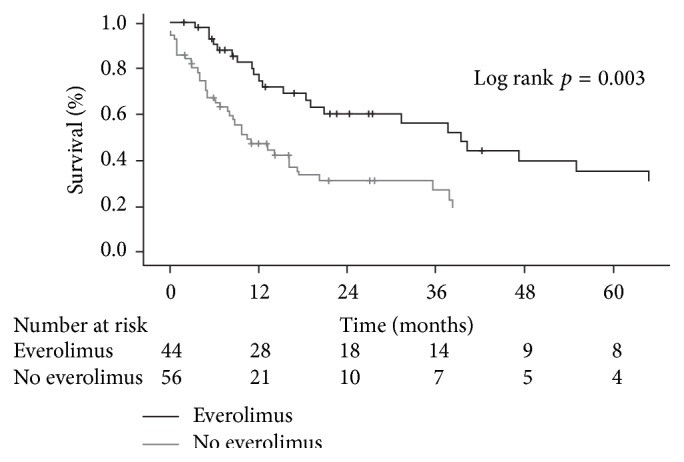
Survival after diagnosis of nonskin malignancy in 39 liver transplant patients according to treatment with everolimus or no everolimus. Reproduced with permission from [71].

Retrospective studies have evaluated survival rates following introduction of everolimus after onset of* de novo* malignancies in liver transplant patients [70–72]. In one small series of 10 patients with posttransplant neoplasm (3 Kaposi's sarcoma, 2 lung cancers, 1 HCC recurrence, 1 HCC-related lung metastasis, 1 diffuse large B-cell lymphoma, and 2 skin cancers), treated with everolimus and followed up for a median of 12.7 months, survival rates were significantly higher than in a group of 14 historical controls with comparable malignancies (100%, 90%, and 72% at months 6, 12, and 24 compared to 50%, 29%, and 14%; *p* = 0.008) [70]. In a larger cohort of 83 patients with* de novo* solid tumors after transplantation for alcoholic liver disease, 38 patients were converted to everolimus (with CNI discontinuation in 25 cases) [71]. Compared to patients who remained on standard CNI therapy (mostly tacrolimus), five-year survival was significantly higher under everolimus (Figure 2). Interestingly, the impact of everolimus was restricted to patients with metastatic disease; no effect was observed in patients with early or intermediate disease. Bilbao et al. have also described good survival rates in a series of 143 liver transplant patients in whom everolimus therapy was started in response to* de novo* malignancy (71.1% at three years) [72]. No noneverolimus control group was included, but for comparison, 157 patients at the center who started everolimus due to renal function deterioration had a three-year survival rate of 83.0%.

### 4.3. Recurrent Hepatocellular Carcinoma

#### 4.3.1. Prevention

Preventing HCC recurrence after transplantation is a particular priority since the cancer is more aggressive than in nontransplanted patients [73] and the prognosis is extremely poor. Extensive preclinical data have pointed to an antitumor effect for mTOR inhibition in HCC [74], and a systematic review of 42 clinical studies involving 3,666 patients receiving a liver transplant for HCC found mTOR inhibition to be associated with a significantly lower rate of HCC recurrence versus CNI therapy (8% versus 13.8%, *p* < 0.001) [75]. This advantage difference was observed despite a lower proportion of HCC within Milan criteria, and a higher rate of microvascular invasions, in the everolimus-treated group. When treatment with everolimus or sirolimus was compared, recurrence rates were lower under everolimus (4.1% versus 10.5%) but this may not be a genuine finding since follow-up time was shorter in the everolimus group (mean 13 versus 30 months with sirolimus) and more patients were within Milan criteria [75].

Three randomized trials in which everolimus were introduced by month 1 after kidney transplantation have reported recurrence rates in the HCC subpopulations [76–78]. In each case, HCC recurrence was numerically less frequent in everolimus-treated patients versus those given standard CNI-based immunosuppression but in two studies the patient numbers were low (<50) [76, 77]. In the H2304 trial, 203 patients were transplanted for HCC [79]. In a* post hoc* analysis of this group after three years' follow-up, recurrence had occurred in 5/136 everolimus-treated patients (3.7%) compared to 8/67 CNI-treated patients (9.7%). These data have been published only in abstract form, with no statistical analysis, but nevertheless represent a relatively large population within a randomized trial and merit further investigation. Also, it is noteworthy that one retrospective study of 21 patients transplanted for HCC outside the Milan criteria and treated with everolimus from week 2 after transplant found the recurrence rate to be 41.3%, compared to 61.3% in a group of 31 CNI-treated controls [80]. Overall, everolimus appears to offer a potential option for reducing the risk for HCC recurrence after liver transplantation, and randomized trials are awaited with interest.

#### 4.3.2. Management

In nontransplanted patients with advanced HCC, early randomized trials have shown encouraging results when everolimus is introduced [81, 82]. One meta-analysis concluded that patients with nontransplant HCC showed a low but significant survival benefit under everolimus monotherapy [83], although this does not apply after failure of sorafenib therapy appears inadequate in advanced cases [84]. Management of recurrent HCC is one of the most frequent reasons for starting everolimus in maintenance liver transplant patients. Published case reports have described good outcomes in nonresectable patients treated with everolimus and sorafenib, although sorafenib side effects are problematic [85–87], but prospective trials are lacking. At this point, no conclusion can be drawn but in nonresectable patients with posttransplant HCC recurrence introduction of everolimus may be helpful, although the drug is not licensed for this indication.

## 5. Heart Transplantation

### 5.1. Prevention of* De Novo* Malignancies

Heart transplant patients experience especially high rates of malignancy [1], possibly due to a greater intensity of immunosuppression. An analysis of 381 patients transplanted at the University of Heidelberg in Germany during 1989 to 2014 investigated an association between development of neoplasms and inclusion of either everolimus or sirolimus in the initial immunosuppressive regimen [13]. During a mean follow-up of 9.7 years, 34.1% of patients developed a neoplasm, most frequently skin cancer (15.2% of patients). Administration of an mTOR inhibitor was associated with a lower risk for malignancy versus no mTOR inhibitor (*p* < 0.001) but significance was lost on multivariate analysis. Notably, however, patients given mTOR inhibition had a lower rate of skin cancer recurrence (*p* = 0.020) and lower mortality related to nonskin malignancies (*p* < 0.001). Studies assessing an effect of everolimus specifically on the rate of* de novo* malignancies are lacking, not least due to the relatively small pool of heart transplant patients treated with everolimus to date.

### 5.2. Management of Posttransplant Malignancy

Malignancy is one of the most frequent indications for introducing everolimus in maintenance heart transplant patients [12, 88]. Data on nonskin malignancies in this setting, however, is virtually absent. Kusuki et al. described the case of a four-year-old heart transplant recipient with diffuse large B-cell lymphoma who was switched from standard CsA to everolimus with low-exposure CsA and given rituximab and combination therapy [89]. The patient achieved an excellent response but the role of everolimus cannot be determined.

**Figure 3:**
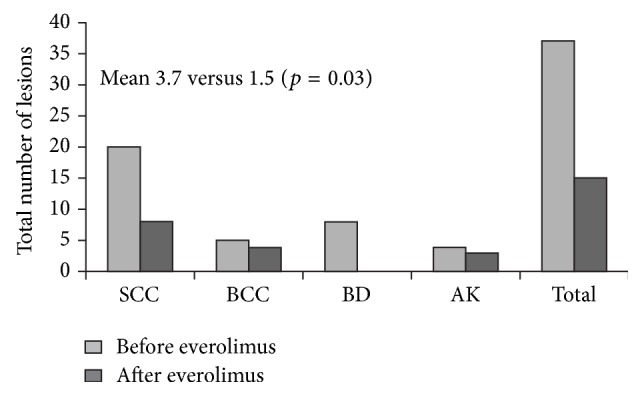
Skin cancers before and after introduction of everolimus in heart transplant recipients. Mean follow-up after start of everolimus was 28 months (range 16 to 37 months) [68]. The number of lesions before everolimus is shown for the preceding 28 months. SCC, squamous cell carcinoma; BCC, basal cell carcinoma; BD, Bowen's disease; AK, actinic keratosis.

Evidence relating to the management of skin cancer after heart transplantation is somewhat more substantial. As in other organ types, skin cancers are the most frequent type of neoplasms after heart transplantation [12] and use of mTOR inhibition appears to delay their recurrence [13]. Euvrard et al. undertook an observational study of 10 patients with multiple recurrent skin tumors and/or fast-growing SCC [68]. All patients were receiving CsA, either as monotherapy or with MMF/azathioprine and/or steroids. Everolimus was introduced, and CsA was stopped in four patients and reduced in the remaining six patients. The number of skin tumors which developed after over a mean of 28 months after starting everolimus was significantly lower than in the preceding 28 months (Figure 3). A case has also been published in which a heart transplant recipient who was developing more than 20 SCC lesions per year was switched from CsA to everolimus, after which the rate slowed to six lesions annually [90]. In this patient, wound healing complications necessitated switch back to CsA, after which skin carcinogenesis returned to the original levels. In another case, a patient receiving tacrolimus, MMF, and steroids developed multiple SCC lesions [91]. Despite excision and repeated topical and photodynamic therapy, more SCCs developed and were increasingly difficult to treat. The patient was switched from tacrolimus to everolimus, resulting in an immediate and profound decrease in both SCC and new actinic keratosis lesions, with the few lesions responding to treatment. Currently, a randomized trial (CERTICOEUR) is comparing the rate of skin cancer recurrence in heart transplant patients receiving everolimus and reduced or discontinued CNI therapy versus standard CNI therapy (NCT00799188).

## 6. Balancing Risks and Benefits

Clearly, any reduction in malignancy risk under everolimus should be balanced by an assessment of risk for graft rejection or drug toxicity. A series of randomized trials in* de novo* kidney transplant patients [39, 92, 93] and in liver transplant patients ~3 months after transplant [78] has indicated that everolimus with reduced CNI therapy offers comparable immunosuppressive efficacy to a standard CNI regimen. It should be noted that these trials generally excluded patients at high immunological risk. Observational studies of everolimus with reduced-exposure CNI from time of liver transplant, one strategy that could be advantageous for risk of HCC, have also shown good efficacy [94, 95] but more robust data are awaited. Randomized studies in which kidney [38, 96, 97], liver [76–78], or heart [45] transplant patients were converted early from a CNI-based regimen to CNI-free everolimus therapy have either maintained efficacy or been associated with an increase in mild episodes of biopsy-proven acute rejection. When considering introduction of everolimus to minimize risk for malignancy, the patient's immunological risk status thus must be carefully considered. In terms of safety, the acute side effects of mTOR inhibitors have conventionally included lymphoceles and delayed wound healing, although under modern concentration-controlled regimens without loading doses there is little evidence either for a marked increase in wound-related complications [98, 99]. Initial concerns about an increase in hepatic artery thrombosis after liver transplantation now appear unfounded [99]. The long-term effects associated with mTOR inhibitors include an increased risk for dyslipidemia, cytopenias, proteinuria, and aphthous stomatitis, which are typically mild and can usually be managed effectively with close monitoring of trough levels and pharmacologic intervention [100, 101].

## 7. Conclusion

Capturing data on malignancy accurately and comprehensively in sufficiently large cohorts of transplant patients over an adequate period is virtually impossible. Registry data can be informative, but necessarily imperfect. Clinical trials, potentially, can assess the risk of recurrence or* de novo* cancers in very high-risk cohorts but cannot be powered to detect difference in rates of* de novo* cancers across typical posttransplant populations. Against this background, it is understandable that the evidence base concerning malignancy risk under everolimus in different types of solid organ transplantation is relatively sparse. Overall, it can be concluded that, in certain settings, notably following onset of skin cancers or Kaposi's sarcoma, or in the prevention and management of HCC after liver transplantation, everolimus appears a promising option to lessen the toll of posttransplant malignancy. Despite the paucity of randomized controlled trials, many kidney transplant centers' response to a diagnosis of posttransplant malignancy under standard CNI therapy includes introduction of an mTOR inhibitor as per KDIGO recommendations [53] with either reduced CNI exposure or CNI discontinuation. This pragmatic approach reflects current uncertainty about the relative contribution of the antioncogenic effects of everolimus versus a lessening or withdrawal of CNI-related prooncogenic effects, a question which is likely to be difficult to answer definitively. Wider recommendations will need to await further data but may need to rely on indirect evidence from high-risk transplant patients or from large-scale analyses such as meta-analyses. Despite the limitations of clinical trials in establishing malignancy risk, scrupulous collection and reporting of data in controlled studies are essential and may contribute to future pooled analyses.

## Figures and Tables

**Table 1:** Case reports of conversion to everolimus for Kaposi's sarcoma after solid organ transplantation.

	Age (years)/type of tx	Location of KS	Time post-tx to switch to everolimus, months	Original IS regimen	Everolimus-based IS regimen	Other intervention for KS	Follow-up (months)	Outcome for KS
Campistol and Schena 2007 [52]	29/kidney	Skin Lung Stomach	24	CsA MMF Steroids	Everolimus Steroids	Doxorubicin	5	Resolution in all locations

Campistol and Schena 2007 [52]	66/kidney	Skin	3	CsA MMF Steroids	Everolimus Steroids	None	4	Resolution

Campistol and Schena 2007 [52]	66/kidney	Not stated	15	Tacrolimus MMF Steroids	Everolimus MMF Steroids	None	4	Resolution

Basu et al. 2011 [55]	55/kidney	Skin Soft palate	6	CsA Azathioprine Steroids	Everolimus Steroids	Leflunomide	36	Resolution

Detroyer et al. 2015 [56]	27/kidney	Skin Liver Lymph nodes	12	Tacrolimus MMF Steroids	Everolimus Steroids	None	9	Resolution at all locations

Lund et al, 2013 [57]	60/lung	Jejunum/ileum	18	CsA MMF Steroids	Everolimus Low CsA Steroids	None	12	Resolution

CsA, cyclosporine; IS, immunosuppressive; KS, Kaposi's sarcoma; MMF, mycophenolate mofetil; tx, transplantation.

**Table 2:** Case reports of conversion to everolimus for nonmelanoma skin cancer after solid organ transplantation.

	Age (years)/type of tx	Type (number) of NMSC	Time post-tx to switch to everolimus, months	Original IS regimen	Everolimus-based IS regimen	Other intervention for NMSC	Follow-up (months)	Outcome for NMSC
Fernández et al. 2006 [51]	70/kidney	SCC (3)	89	CsA	Everolimus^a^	None	Mean 6.5 months	Existing lesions improved No recurrence

Fernández et al. 2006 [51]	69/kidney	SCC (1), BCC (1)	65	Tacrolimus	Everolimus^a^	Excision	Mean 6.5 months	No recurrence

Fernández et al. 2006 [51]	64/kidney	SCC (6)	116	CsA MMF	Everolimus^a^ MMF	None	Mean 6.5 months	Existing lesions resolved No recurrence

Fernández et al. 2006 [51]	70/kidney	SCC (17), BCC (1)	206	Tacrolimus	Everolimus^a^	Excision	Mean 6.5 months	No recurrence

Fernández et al. 2006 [51]	67/kidney	SCC (1), BCC (2)	130	CsA MMF	Everolimus^a^ MMF	None	Mean 6.5 months	Existing lesions resolved No recurrence

Fernández et al. 2006 [51]	69/kidney	SCC (2), BCC (1) & actinic keratosis (1)	178	CsA	Everolimus^a^	Excision	Mean 6.5 months	No recurrence

Pascual et al. 2006 [66]	64/kidney	Recurrent cutaneous neoplasms (5)	Not stated	CsA MMF Steroids	Everolimus MMF Steroids	Excision	9	No recurrence

Alter et al. 2014 [67]	71/kidney	SCC (4), BD (2)	36	CsA MMF Steroids	Everolimus MMF Steroids	Excision, curettage, and photodynamic therapy	24 months	SCC (2)

Alter et al. 2014 [67]	49/heart	BCC (2), BD (1)	180	CsA AZA Steroids	Everolimus CsA	Excision, curettage, and photodynamic therapy	24 months	BCC (2)

Alter et al. 2014 [67]	44/lung	SCC (3), BD (3)	264	CsA AZA Steroids	Everolimus CsA Steroids	Excision, curettage, and photodynamic therapy	24 months	No lesions (12 months)

Alter et al. 2014 [67]	62/kidney	SCC (1), BCC (1), BD (4)	66	AZA Steroids	Everolimus Steroids	Excision, curettage, and photodynamic therapy	24 months	No lesions

Alter et al. 2014 [67]	57/heart	BD (2)	120	CsA AZA Steroids	Everolimus CsA Steroids	Excision, curettage, and photodynamic therapy	24 months	No lesions

^a^Use of steroids before or after conversion to everolimus was not stated.

AZA, azathioprine; BCC, basal cell carcinoma; BD, Bowen's disease; CsA, cyclosporine; IS, immunosuppressive; MMF, mycophenolate mofetil; NSMC, nonmelanoma skin cancer; SCC, squamous cell carcinoma; tx, transplantation.

## Abbreviations

BCC: Basal cell carcinoma
CNI: Calcineurin inhibitor
CsA: Cyclosporine
HCC: Hepatocellular carcinoma
MMF: Mycophenolate mofetil
mTOR: Mammalian target of rapamycin
NMSC: Nonmelanoma skin cancer
PTLD: Posttransplant lymphoproliferative disease
SCC: Squamous cell carcinoma
TGF-*β*: Transforming growth factor beta
VEGF: Vascular endothelial growth factor.
